# Supplementary material for: Factors Associated with Cardiac/Pericardial Injury among Blunt Injury Patients: A Nationwide Study in Japan
Source: J Clin Med. 2022 Aug 3;11(15):4534. doi: 10.3390/jcm11154534 (PMC9369737; doi:10.3390/jcm11154534)
Supplement: Supplementary file 1 [file jcm-11-04534-s001.zip › jcm-1821938-supplementary.pdf]

Table S1. Abbreviated Injury Scale (AIS) code for cardiac/pericardial injury.

Injury Site	AIS Code	Description of Injury
Heart	441099.1	Heart (Myocardium), NFS
	441002.1	Contusion (hematoma), NFS
	441004.1	Contusion, minor, patients presenting with dysrhythmia, wall mation abnormality, other ECG changes not related to coronary artery disease
	441006.4	Contusion, major, this diagnosis must be substantiated, e.g., by surgery, autopsy, ejection fraction <25% absent coronary artery disease
	441008.3	Lacelation, NFS
	441010.3	Laceration, no perforation, no chamber involvement
	441012.5	Laceration, perforation (ventricular or atrial with or without tamponade)
	441014.6	Laceration, perforation, complex or ventricular rupture
	441016.6	Laceration, perforation, multiple lacerations; >50% tissure loss of a chamber
	441018.6	Avulsion
	441200.5	Intracardiac valve laceration (rupture)
	441300.5	Intraventricular or interatrial sptum laceration (rupture)
Pericardium	441699.2	Pericardium, NFS
	441602.2	Laceration (rupture)
	441604.3	Injury with tamponade without heart injury
	441606.5	Herniation of heart

AIS—Abbreviated Injury Scale; ECG—echocardiogram; NFS—not further specified.

Table S2. AIS code for concomitant thoracic injury.

Thoracic Aortic Injury	Tracheal Injury	Esophageal Injury	Pulmonary Contusion	Rib Fracture	Sternal Fracture	Clavicle Fracture	Hemothorax / Pneumothorax	Thoracic Spinal Injury
420202.4	442602.3	440802.2	441402.3	450210.2	450804.2 450802.1	752200.2	Thoracic aorta	630402.2
420204.5	442604.3	440804.3	441406.3	450211.3	Sternum, contusion (exclusion)		420218.6	630404.2

Thoracic Aortic Injury	Tracheal Injury	Esophageal Injury	Pulmonary Contusion	Rib Fracture	Sternal Fracture	Clavicle Fracture	Hemothorax / Pneumothorax	Thoracic Spinal Injury
420206.4	442606.3	440806.3	441410.4	450212.1	450899.1 Sternum, NFS (exclusion)		Lung	630406.2
420208.4	442608.4	440808.4	441414.3	450214.3			441422.5	630408.3
420210.5	442610.5	440810.5	441416.3	450220.2			441424.5	630410.2
420299.4	442612.4	440899.2	441424.5	450222.3			441438.5	640400.3
420212.5	442614.4		441418.4	450230.3			Thoracic cavity	630412.2
420216.5	442616.5		441420.4	450232.4			441440.5	630414.3
420218.6	442699.3		441422.5	450240.4			441458.5	630499.2
			441426.5	450242.5			441460.5	640400.5
			441430.3	450250.3			442202.3	640401.3
			441432.3	450252.4			442210.5	640402.3
			441434.4	450260.3			Rib cage	640404.3
			441436.4	450262.3			450211.3	640406.3
			441438.5	450264.4			450214.3	640408.3
			441440.5	450266.5			450222.3	640410.4
			441442.5	450202.1 rib cage, contusion (exclusion)			450232.4	640412.4
			441450.4	450299.1 rib cage, NFS (exclusion)			450242.5	640414.4
			441452.4				450252.4	640416.4
			441454.4					640418.4
			441456.5					640420.5
			441458.5					640422.5
			441460.5					640424.5
			441462.5					640426.5
			441499.3					640428.5
								640442.5
								640444.5
								640446.5
								640448.5

Thoracic Aortic Injury	Tracheal Injury	Esophageal Injury	Pulmonary Contusion	Rib Fracture	Sternal Fracture	Clavicle Fracture	Hemothorax / Pneumothorax	Thoracic Spinal Injury
								640450.5
								640460.5
								640462.5
								640464.5
								640466.5
								640468.5
								640478.1
								640484.1
								650400.2
								650402.2
								650403.3
								650404.2
								650409.2
								650410.2
								650412.3
								650416.2
								650418.2
								650420.2
								650422.3
								650424.3
								650426.3
								650430.2
								650432.2
								650434.3
								650499.2

AIS—Abbreviated Injury Scale; NFS—not further specified.
